# Nanoparticle treatment of maize analyzed through the metatranscriptome: compromised nitrogen cycling, possible phytopathogen selection, and plant hormesis

**DOI:** 10.1186/s40168-020-00904-y

**Published:** 2020-09-09

**Authors:** Wouter M. A. Sillen, Sofie Thijs, Gennaro Roberto Abbamondi, Roberto De La Torre Roche, Nele Weyens, Jason C. White, Jaco Vangronsveld

**Affiliations:** 1 Centre for Environmental Sciences, Hasselt University, Agoralaan Building D, 3590 Diepenbeek, Belgium; 2 Institute of Biomolecular Chemistry, National Research Council of Italy, Via Campi Flegrei 34, Pozzuoli, 80078 Napoli, Italy; 3 Department Analytical Chemistry, Connecticut Agricultural Experiment Station, 123 Huntington Street, New Haven, CT USA; 4 Department of Plant Physiology, Faculty of Biology and Biotechnology, Maria Curie-Sklodowska University, Lublin, Poland

**Keywords:** Plant microbiome, Maize, Silver nanoparticles, Rhizosphere, Metatranscriptome, Phytopathogens

## Abstract

**Background:**

The beneficial use of nanoparticle silver or nanosilver may be confounded when its potent antimicrobial properties impact non-target members of natural microbiomes such as those present in soil or the plant rhizosphere. Agricultural soils are a likely sink for nanosilver due to its presence in agrochemicals and land-applied biosolids, but a complete assessment of nanosilver’s effects on this environment is lacking because the impact on the natural soil microbiome is not known. In a study assessing the use of nanosilver for phytopathogen control with maize, we analyzed the metatranscriptome of the maize rhizosphere and observed multiple unintended effects of exposure to 100 mg kg^−1^ nanosilver in soil during a growth period of 117 days.

**Results:**

We found several unintended effects of nanosilver which could interfere with agricultural systems in the long term. Firstly, the archaea community was negatively impacted with a more than 30% decrease in relative abundance, and as such, their involvement in nitrogen cycling and specifically, nitrification, was compromised. Secondly, certain potentially phytopathogenic fungal groups showed significantly increased abundances, possibly due to the negative effects of nanosilver on bacteria exerting natural biocontrol against these fungi as indicated by negative interactions in a network analysis. Up to 5-fold increases in relative abundance have been observed for certain possibly phytopathogenic fungal genera. Lastly, nanosilver exposure also caused a direct physiological impact on maize as illustrated by increased transcript abundance of aquaporin and phytohormone genes, overall resulting in a stress level with the potential to yield hormetically stimulated plant root growth.

**Conclusions:**

This study indicates the occurrence of significant unintended effects of nanosilver use on corn, which could turn out to be negative to crop productivity and ecosystem health in the long term. We therefore highlight the need to include the microbiome when assessing the risk associated with nano-enabled agriculture.

Video Abstract

## Background

The development and application of nanotechnology has impacted numerous disciplines across a broad array of sectors, including communications, medicine, energy, and agriculture. In order to ensure the sustainable and optimal application of these products and materials, it is important to thoroughly understand both the risks and benefits associated with their use. However, the diverse and highly complex interplay of biotic and abiotic factors in environments such as soil serve as a major confounding factor to efforts designed to understand the potential negative or positive impacts associated with nanomaterials and nano-enabled products. Studies in artificial in vitro conditions or with a single species cannot be easily extrapolated to natural multispecies conditions. The rhizosphere or soil root-zone is a prime example of such a complex environment. Both prokaryotic and eukaryotic microorganisms reside in root-affected soil, and these groups of microbes can interact directly or indirectly with each other or with the plant in ways that may be beneficial, antagonistic, or neutral [1–3]. However, given the importance of the rhizosphere to both agriculture and important ecosystem services such as nutrient cycling (e.g., nitrogen fixation), a thorough understanding of the range of potential impacts of nanomaterial exposure in this complex environment is of critical importance [4].

Silver nanoparticles are among the most widely used nanomaterials, largely due to their known antimicrobial properties which make them highly valuable in textiles, food packaging, cosmetics, medicinal products, and many other applications [5, 6]. This is illustrated by the 379 consumer products, such as textiles, food packaging, cosmetics, and medicinal applications listed in The Nanodatabase as containing nanosilver as of 2020 [7]. Consequently, the number of studies addressing the environmental impact of nanosilver has increased significantly during the last 5 years. As alluded to above, investigations in soil under realistic exposure scenarios, including multispecies environments, are rare. A number of prominent researchers in this field have noted that the next important step in nanomaterial ecotoxicology after hazard-based screening studies is to increase the environmental realism of exposure by approximating conditions closer to the field situation [8]. This obviously applies to nanosilver, as its molecular toxicity and its effects on various species need to be evaluated under realistic exposure scenarios that can also account for other unforeseen abiotic and biotic interactions that may occur. The rhizosphere of important crop species such as maize are prone to nanosilver exposure given that agricultural soils are an important sink for engineered nanomaterials through the application of nano-enabled agrochemicals and nanomaterial-containing biosolids [9–11]. In this root-soil interface, nanosilver could impact both this economically important crop species and the microbial network residing in its vicinity. Given the known antimicrobial properties of nanosilver, understanding the impact of this material on phytopathogen activity, the rhizosphere microbiome, and on the cycling and availability of nutrients [3] is of particular concern.

In this study, we grew maize to maturity in nanosilver-containing soil and analyzed a range of endpoints, including plant biomass, tissue element content, and the rhizosphere metatranscriptome. The primary benefit of a metatranscriptomic analysis is that it offers an opportunity to investigate all active organisms in the corn rhizosphere as a function of nanosilver exposure. Specifically, the microbial community structure and the expressed genes of both prokaryotic and eukaryotic microorganisms can be studied, as well as the expressed genes from maize roots themselves. This allows to provide new information on nanosilver’s direct and indirect effects on biota at the individual and community level, as well as on important processes in the complex soil ecosystem. Hence, this creates a study framework where multiple interactors of a natural system are studied, getting a step closer to the holistic view of nanosilver toxicology in a natural setting which is alluded to above. Because of the importance and ubiquity of maize growth, the data provided on this model crop has relevance to both nanotoxicology and to nano-enabled agriculture. Also, similar mechanisms of response can be expected in the rhizosphere of other crops, extending the results of this study beyond this single species.

## Results and discussion

### Mixed responses by bacteria to nanosilver are likely determined by silver defense systems

**Fig. 1:**
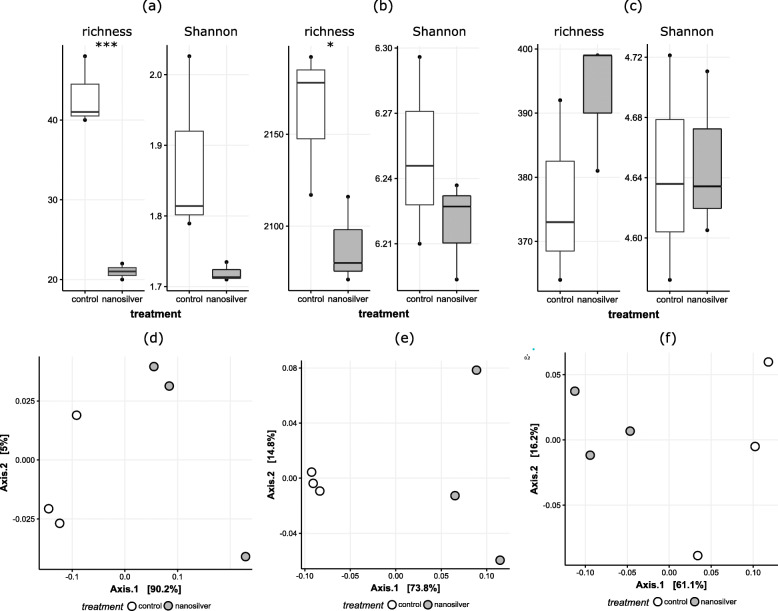
Alpha and beta diversity of maize rhizosphere archaea, bacteria and fungi. Richness and Shannon diversity index for (**a**) archaea, (**b**) bacteria, and (**c**) fungi; and PCoA plot based on Bray-Curtis dissimilarities for (**d**) archaea, (**e**) bacteria, and (**f**) fungi for maize rhizosphere soil communities with (grey) or without (white) exposure to 100 mg kg^−1^ silver nanoparticles (20 nm) in soil, based on the metatranscriptome. Statistically significant differences for the alpha diversity measures are indicated on top of the graph: *p < 0.05, **p < 0.01, ***p < 0.001

**Fig. 2:**
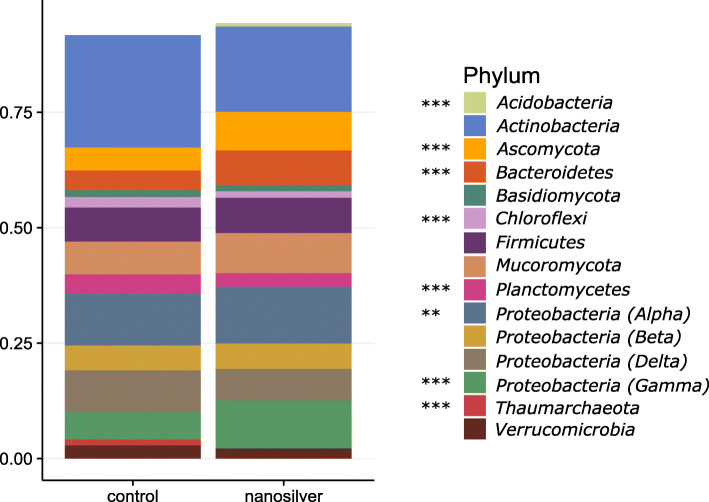
Maize rhizosphere microbiome community composition at phylum level. Microbiome community composition at the phylum level in maize rhizosphere soil with or without 100 mg kg^−1^ silver nanoparticles (20 nm), based on the metatranscriptome. Relative abundance is shown and only phyla making up more than 1% of the total community are taken into account. Phyla with statistically significant differences between control and nanosilver-exposed conditions are indicated next to the legend: *p < 0.05, **p < 0.01, ***p < 0.001

**Fig. 3:**
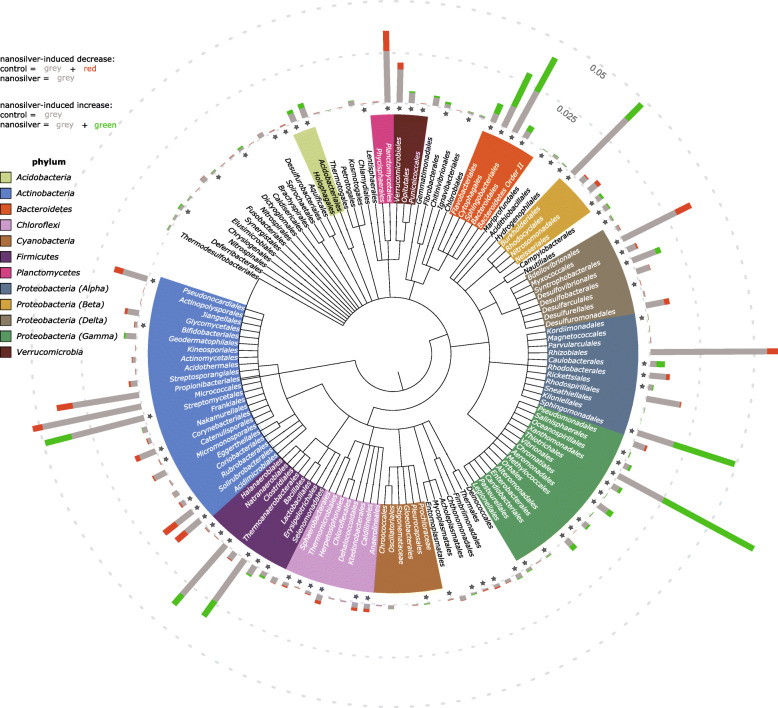
Maize rhizosphere bacteria community composition at order level. Community composition of bacteria at the order level in maize rhizosphere soil with or without 100 mgkg^−1^ silver nanoparticles (20 nm), based on the metatranscriptome. Bar graphs indicate abundance, with the effect of nanosilver exposure shown in green (increased compared to control) or red (decreased compared to control). Scale reference is indicated as dotted lines. Statistical significance (*p* < 0.05) of the nanosilver effect is indicated by a star

Nanosilver’s antimicrobial properties are expected to affect the microbiome, and the bacterial community composition in the maize rhizosphere does clearly reflect this. Indeed, alpha diversity analysis indicates a significant decrease in richness (Fig. 1b). However, not all bacteria displayed a similar response of decreasing in abundance. This can already be deduced from the PCoA-plot (Fig. 1e) as the communities of the control and nanosilver-exposed treatments appear to be separated, although the statistical significance of this could not be verified (ANOSIM *p* = 0.1). Nonetheless, taxonomic analysis of the bacterial communities clearly indicates already at the phylum level that different bacterial groups responded differently to nanosilver exposure (Fig. 2). While *Chloroflexi* and *Planctomycetes* abundance decreased significantly (*p* < 0.05) in the active rhizosphere microbiome, *Bacteroidetes*, *Alphaproteobacteria*, *Gammaproteobacteria*, and *Acidobacteria* were all increased in response to nanosilver exposure. Other groups, such as *Actinobacteria* and *Deltaproteobacteria*, showed a trend of decrease, although the effect was not statistically significant. Figure 3 indicates that also within these non-significantly affected phyla, there are bacterial orders, e.g., *Acidimicrobiales*, *Solirubrobacterales,* and *Pseudonocardiales*, that show significant decreases due to nanosilver exposure. This lower taxonomic level also indicated that important bacteria orders such as *Fimbriimonadales*, *Chthonomonadales*, *Verrucomicrobiales*, *Myxococcales*, *Synthrophobacterales*, and *Rhodobacterales* showed significant decreases.

The impact of nanosilver on the bacterial community is also illustrated by the functional characterization of the metatranscriptome. Table 1 shows the transcripts with a significantly (*p* < 0.05) different abundance in rhizosphere soil. Gene transcripts of P-type ATPases, Cu^+^-exporting ATPases, and Cu(I)/Ag(I) efflux system proteins showed increases in abundance. Some of these are related to bacterial groups that increased in abundance due to nanosilver, such as *Proteobacteria*, *Bacteroidetes*, and *Acidobacteria*, as well as to bacterial groups that decreased due to nanosilver, such as *Actinobacteria* and *Planctomycetes*. Hence, regardless of their final decrease or increase in abundance in response to nanosilver exposure, diverse bacterial groups initiate defense responses. However, the groups that thrive under nanosilver exposure display increased abundances of additional important genes such as cation efflux system CzcA, copper-resistance protein CopA, and Copper-resistance protein K. These additionally expressed genes could explain the advantage that these bacterial groups have over others which do not express these genes and decrease in overall abundance. Such copper and other metal resistance genes seem to be similar and evolutionarily linked to silver resistance systems such as the *sil* silver-resistance system [12]. The *sil* system often is associated with the incompatibility group H1 (IncH1) plasmid pMG101, which is transferred horizontally and confers resistance to Gram-negative but not to Gram-positive bacteria [13, 14]. This suggests that horizontal gene transfer of such plasmid-borne resistance systems could play a vital role in determining the community shifts taking place in soil under nanosilver exposure. These mechanisms may explain the observed success of Gram-negative groups such as *Alphaproteobacteria*, *Gammaproteobacteria*, and *Bacteroidetes* in nanosilver-exposed soil. Groups that cannot rely on the defensive action of this silver resistance system will be at a disadvantage, and if these organisms are plant-beneficial microbes, then plants could also be negatively affected.

**Table 1:** Functional composition of the metatranscriptome. Metatranscriptome genes annotated to InterPro2GO and KEGG databases with significantly different abundances between control conditions and nanosilver-exposed (100 mg kg^−1^ soil) conditions in maize rhizosphere soil. Taxonomic origin of the genes is given at a general level, as well as the lowest possible level

	Database reference	Database entry	Taxonomic origin					Lowest level taxonomic assignment	Log2 fold change	Base level: mean number of reads
			Archaea	Bacteria	Maize	Fungi	Other			
Higher incidence in AgNP-conditions	IPR021604	Copper resistance protein K		x				Burkholderiales	− 3.59	34.7
	IPR027256	P-type ATPase, subfamily IB	x	x				Actinobacteria, Proteobacteria, Bacteroidetes, Thaumarchaeota, Euryarchaeota	− 1.30	122.4
	IPR004763	Cation efflux system CzcA/CusA/SilA/NccA/HelA/CnrA		x				Bacteroidetes, Proteobacteria, Acidobacteria	− 2.53	29.3
	IPR006376	Copper-resistance protein CopA		x				Sphingomonadales, Xanthomonadales	− 2.26	5.3
	K07787	Cu(I)/Ag(I) efflux system membrane protein CusA/SilA		x				Bacteroidetes, Proteobacteria, Acidobacteria	− 2.53	29.0
	K07798	Membrane fusion protein, Cu(I)/Ag(I) efflux system		x				Proteobacteria, Acidobacteria, Nitrospira, Planctomycetes	− 1.81	9.3
	K17686	Cu^+^-exporting ATPase	x	x				Proteobacteria, Actinobacteria, Thaumarchaeota	− 1.25	83.5
	IPR001806	Small GTPase superfamily		x				Xanthomonadales	− 0.64	171.1
	IPR005294	ATPase, F1 complex, alpha subunit		x				Bacteria general	− 1.23	76.2
	GO:0005634	Nucleus			x	x	x	Eukaryote general	− 0.73	52.0
	ko04121	Ubiquitin system			x	x	x	Eukaryote general	− 0.59	218.9
	ko04144	Endocytosis			x	x	x	Eukaryote general	− 0.62	126.7
	ko01009	Protein phosphatase and associated proteins			x	x	x	Eukaryote general	− 0.42	229.1
	IPR005093	RNA-directed RNA polymerase beta-chain					x	Leviviridae (Bacteriophage)	− 3.12	113.8
	K09872	aquaporin PIP			x			NA	− 1.13	1.8
	ko04075	Plant hormone signal transduction			x			NA	− 2.37	12.1
	ko00940	Phenylpropanoid biosynthesis			x			NA	− 1.26	49.7
	IPR005922	Phenylalanine ammonia-lyase			x			NA	− 2.98	3.1
	K01188	Beta-glucosidase			x			NA	− 2.15	6.2
	IPR015655	Protein phosphatase 2C family			x			NA	− 1.27	17.4
	IPR001246	Lipoxygenase, plant			x			NA	− 2.57	2.8
	K04733	Interleukin-1 receptor-associated kinase 4			x			NA	− 1.77	6.2
	IPR000864	Proteinase inhibitor I13, potato inhibitor I			x			NA	− 1.89	1.7
	IPR001929	Germin			x			NA	− 2.21	3.2
	ko00480	Glutathione metabolism		x	x			Proteobacteria, Actinobacteria	− 0.48	119.1
	IPR003855	Potassium transporter			x			NA	− 1.89	4.7
	IPR009716	Ferroporti-1			x			NA	− 1.77	1.3
	K08176	MFS transporter, PHS family, inorganic phosphate transporter			x			NA	− 1.50	2.5
	K00695	Sucrose synthase			x			NA	− 1.90	5.2
	K10592	E3 ubiquitin-protein ligase HUWE1			x			NA	− 1.58	4.0
	IPR005150	Cellulose synthase			x			NA	− 1.93	3.6
	IPR016461	O-methyltransferase COMT-type			x			NA	− 1.63	7.9
	IPR018167	S-adenosylmethionine decarboxylase subgroup			x			NA	− 2.09	2.2
	K02132	F-type H+-transporting ATPase subunit alpha		x	x	x		Streptomyces, Leotiomyceta	− 1.38	22.5
	K02262	Cytochrome c oxidase subunit 3		x	x	x		Streptomyces, Leotiomyceta	− 1.78	11.8
	K03936	NADH dehydrogenase (ubiquinone) Fe-S protein 3		x	x	x		Streptomyces, Leotiomyceta	− 1.56	2.9
	IPR001128	Cytochrome P450		x	x	x		Streptomyces, Leotiomyceta	− 0.84	36.5
	GO:0005739	Mitochondrion			x	x		Sordariomycetes	− 1.76	6.2
	ko04110	Cell cycle			x	x		Leotiomyceta	− 0.95	44.0
	IPR012220	Glutamate synthase, eukaryotic			x	x		Sordariomycetes	− 1.85	5.4
	ko00199	Cytochrome P450		x	x			Streptomyces	− 1.39	8.7
	GO:0004872	Receptor activity		x	x			Streptomyces	− 1.52	19.5
	IPR00136	Glycoside hydrolase, family 1		x	x			Streptomyces, Sphingomonadales	− 2.00	13.2
	K00066	GDP-mannose 6-dehydrogenase		x				Pseudomonadales	− 1.63	7.6
	IPR031148	Plexin family		x				Streptomyces	− 2.47	7.8
	IPR001795	RNA-directed RNA polymerase, luteovirus					x	Luteoviridae	− 1.92	13.5
Lower incidence in AgNP-conditions	IPR010946	Geranylgeranylglyceryl phosphate synthase	x					Nitrosphaeraceae	2.07	24.2
	IPR005938	AAA ATPase, CDC48 family	x					Nitrosphaeraceae	0.85	49.4
	IPR002386	Amicyanin/Pseudoazurin	x					Nitrosphaeraceae	1.38	20.5
	IPR005848	Urease, alpha subunit	x					Nitrosphaeraceae	1.55	34.2
	IPR000812	Transcription factor TFIIB	x					Nitrosphaeraceae	1.54	29.7
	IPR024656	Ammonia monooxygenase, subunit A, archaeal	x					Archaea general	1.69	42.7
	K04080	molecular chaperone IbpA		x				Rhizobiales	1.09	47.2
	IPR026042	Stress response protein YjbJ		x				Alpha- & Gammaproteo, Bacteroidetes	1.17	183.5
	IPR031107	Small heat shock protein HSP20		x				Rhizobiales, Chloroflexi, Actinobacteria	0.80	284.3
	IPR001189	Manganese/iron superoxide dismutase		x				Bacteria general	0.61	75.2
	IPR001287	Nitrite reductase, copper-type	x					Nitrosphaeraceae	1.21	29.2
	K02518	Translation initiation factor IF-1		x				Actinobacteria	1.01	50.8

### Increase in fungal abundance, including potentially phytopathogenic groups

**Fig. 4:**
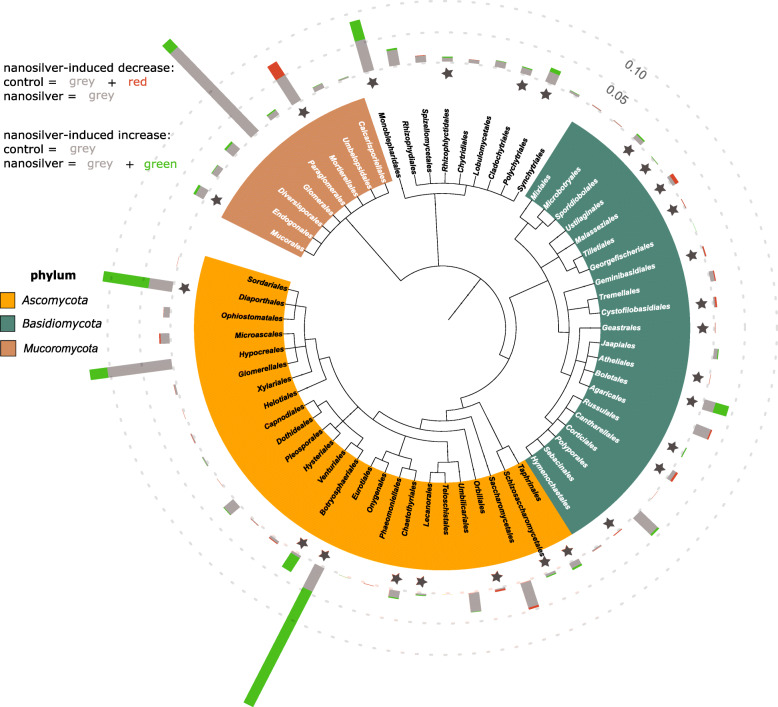
Maize rhizosphere fungi community composition at order level. Community composition of fungi at the order level in maize rhizosphere soil with or without 100 mg kg^−1^ silver nanoparticles (20 nm), based on the metatranscriptome. Bar graphs indicate abundance, with the effect of nanosilver exposure shown in green (increased compared to control) or red (decreased compared to control). Scale reference is indicated as dotted lines. Statistical significance (*p* < 0.05) of the nanosilver effect is indicated by a star

Next to bacteria, fungi are also susceptible to nanosilver toxicity. However, the trend of increase in richness already indicates that at least part of the fungal community benefited from nanosilver addition to the soil (Fig. 1c). Fungal community shifts are hinted at by the PCoA plot (Fig. 1f), although statistical significance could not be inferred (ANOSIM *p* = 0.1). Community composition at the phylum level showed a significant increase in abundance of *Ascomycota*, which appear to benefit from the presence of nanosilver in soil (Fig. 2). Figure 4 reveals more detail as several fungal orders were significantly altered in abundance, but still most fungal groups displayed an increase in abundance. Orders of *Basidiomycota* reacted variably to nanosilver, with positive and negative responses equally divided. *Ascomycota* showed a different pattern, with nearly uniform increases associated with nanosilver exposure. Importantly, the orders *Diaporthales*, *Eurotiales*, and *Botryosphaeriales* experienced the greatest positive response to nanosilver exposure. These three orders all contain genera with well-known phytopathogens; significantly greater abundance of these groups within the nanosilver-exposed rhizosphere microbiome is of concern. Certain species from the genus *Diplodia*, belonging to the *Botryosphaeriales*, are causal agents of stalk and ear rot in maize, and this genus showed a 5.5-fold increase in relative abundance under nanosilver exposure. In the *Eurotiales*, species from the genera *Aspergillus* and *Penicillium* can cause maize ear rot, and these genera displayed 87% increase and a 6.25-fold increase under nanosilver exposure, respectively. The genus *Valsa*, showing a 3.15-fold increase under nanosilver exposure, belongs to the *Diaporthales*, and certain species from this genus are well-described phytopathogens, although not known for maize infection. Overall, such an increase in abundance of possible phytopathogens is not expected to be directly caused by nanosilver but rather is likely to be induced by an indirect mechanism involving interactions between microorganisms.

### Disruption of natural biocontrol as a cause of phytopathogen increase

**Fig. 5:**
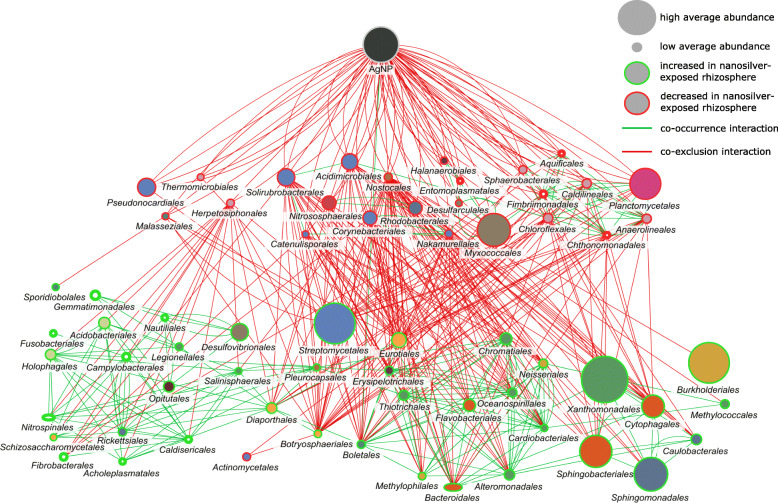
Co-occurrence and co-exclusion network of the maize rhizosphere microbial community. Created from metatranscriptome microbial orders abundance data. Pearson and Spearman correlation, mutual information, Bray-Curtis, and Kullback-Leibler dissimilarity were used, and only statistically significant interactions (*p* < 0.01) are shown. Node size is related to average abundance, node border color indicates nanosilver effect (green = increase, red = decrease), and edge color shows interaction type (green = co-occurrence, red = co-exclusion)

Network analysis based on co-occurrence and co-exclusion interactions enabled differentiation between groups which play an important role in the rhizosphere microbiome by influencing other groups from those that act more individually. As shown in Fig. 5, nanosilver was almost exclusively involved in negative interactions, the exception being a positive interaction with *Boletales*. Therefore, nanosilver exposure was not associated with the direct promotion of abundance of nearly any species. Hence, the cause of the increased abundance of the groups noted above after nanosilver exposure is clearly more complex. Several of the orders with significantly decreased abundance as shown by the taxonomic analysis do appear as direct negative interactors with nanosilver in the network analysis. Phyla belonging to this group include *Actinobacteria*, *Aquificae*, *Armatimonadetes*, *Basidiomycota*, *Chloroflexi*, *Cyanobacteria*, *Firmicutes*, *Planctomycetes*, *Proteobacteria*, *Tenericutes*, and *Thaumarchaeota*. It is noteworthy that *Actinobacteria* are decreased as this group are well-known producers of antibiotics [15] and hence, are involved in negative interactions with numerous species. Of the orders with negative interaction with nanosilver, *Actinobacteria* orders such as *Solirubrobacterales*, *Acidimicrobiales*, *Corynebacteriales*, *Nakamurellales*, *Bifidobacteriales*, and *Catenulisporales* were among the most abundant with the highest number of negative interactions (negative degree). These negative interactions involved a large number of orders which displayed an increased abundance in the nanosilver-exposed rhizosphere microbiome. Hence, according to this network, the increased abundance of certain groups upon nanosilver exposure was not a direct consequence of nanosilver. Rather, these groups benefited from the negative effect of nanosilver on certain other groups which are involved in negative interactions with them. A wide range of microbial phyla were included in this positively affected group. Among these, most notable are the fungal orders which harbor well-known fungal pathogens, as described previously: *Diaporthales*, *Eurotiales*, and *Botryosphaeriales*. This negative interaction of certain microorganisms with phytopathogens suggests the presence of natural biocontrol. When evaluating the role of the rhizosphere microbiome in plant health, biocontrol of phytopathogens is always of high importance [16, 17]. Certain microorganisms can directly, through antibiotic metabolite production, or indirectly, through competition for resources, antagonize phytopathogen populations [18]. Our data suggest that nanosilver in soil has the capacity to promote potentially phytopathogenic fungi, possibly by interfering with this inherent control system afforded by members of the rhizosphere microbiome. Plants such as maize are known to exhibit an age-related resistance [19] that is potentially correlated with the rhizosphere microbiome changes that occur over time [20]. A possible selection of a disease-suppressive microbial community may be prevented by the community alterations from nanosilver exposure. For example, *Actinobacteria* are often increasingly abundant in the rhizosphere of maturing plants [21] and are widely recognized for their biocontrol potential through the production of bioactive compounds [15]. The majority of biocontrol studies with *Actinobacteria* have focused on Streptomyces, illustrated by the fact that this group is responsible for over 80% of all known antibiotics of actinobacterial origin [22]. In the current study, *Streptomycetales* are the only *Actinobacteria* with increased abundance in the rhizosphere under nanosilver exposure. As such, only non-streptomycete *Actinobacteria* could be involved in the phytopathogen proliferation. Next to *Streptomycetales*, other *Actinobacteria* are also recognized as a valuable topic for biocontrol-research, as has been highlighted for *Actinomycetales* [23], and our results suggest that several other *Actinobacteria* orders could be involved here. This includes orders within the class *Actinobacteria*, e.g., *Corynebacteriales* and *Bifidobacteriales*, and orders in other classes, such as *Rubrobacterales* and *Solirubrobacterales*. Another interesting group of bacteria involved in the negative interaction with phytopathogens are *Myxococcales*. These bacteria are also known for their production of a wide range of antibiotics and lytic enzymes, and are often considered to be important to biocontrol [24, 25]. *Rhodobacterales* are another well-studied group of biocontrol agents. All of these bacterial groups could play an important role in limiting maize phytopathogen activity, and importantly, are *all* reduced by nanosilver exposure. These groups may potentially be joined by other bacteria that can negatively impact phytopathogen activity but whose role is unknown due to a lack of culturability. Biocontrol agents are of high value in both natural ecosystems and in agricultural crop production, and nanosilver-induced disturbance of this native protection system could have significant ecologic and economic consequences.

### Archaea decrease and their involvement in nitrogen cycling is confounded

**Fig. 6:**
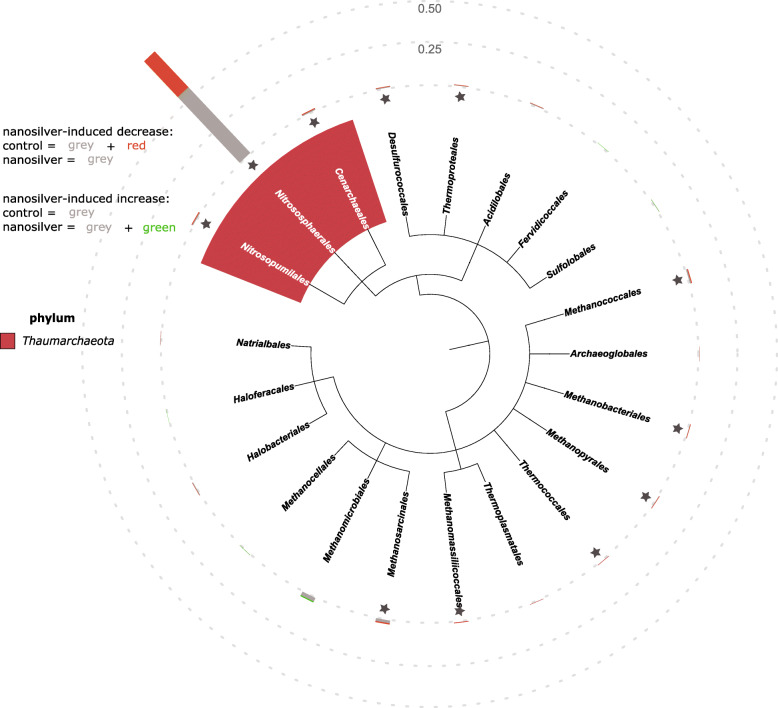
Maize rhizosphere archaea community composition at order level. Community composition of archaea at the order level in maize rhizosphere soil with or without 100 mg kg^−1^ silver nanoparticles (20 nm), based on the metatranscriptome. Bar graphs indicate abundance, with the effect of nanosilver exposure shown in green (increased compared to control) or red (decreased compared to control). Scale reference is indicated as dotted lines. Statistical significance (*p* < 0.05) of the nanosilver effect is indicated by a star

Archaea possess a number of properties which could give them an advantage under nanosilver-stress. These organisms have tetraether-linked membrane lipids as the main lipid component in their cell membrane, sometimes organized into a monolayer which increases the impermeability to metals [26]. Also, archaea are known to outcompete bacteria under chronic energy stress conditions [27], and within the archaeal domain, genes for the metabolism, resistance, and detoxification of metals are reported to be widespread [28]. These features, along with other properties, give an advantage to archaea in responding to metal exposure. Indeed, archaea showed increased transcript abundance of two metal-exporting proteins, i.e., select P-type ATPase and Cu^+^-exporting ATPase (Table 1). Nonetheless, in spite of these protective mechanisms, archaea in the rhizosphere soil decreased in abundance upon nanosilver exposure, as is evident from a significant decrease in richness (Fig. 1a) and decreases in abundance of individual taxa (Figs. 2 and 6). Important archaeal genes such as urease and ammonia monooxygenase exhibited decreased transcript abundance, suggesting that the negative impact of nanosilver on this group could compromise nitrogen cycling. The contribution of ammonia-oxidizing archaea (AOA) to nitrification in soil has been discovered somewhat recently [29, 30], with its relative importance to ammonia oxidizing bacteria (AOB) varying between soil types [31]. Some studies have focused on the effect of nanosilver on nitrification, but have primarily found an impact on bacterial ammonia oxidation [32, 33]. The general presence of archaea has been examined in several studies with metal exposure, with results of different degrees of increased and decreased archaeal abundance [34–37]. Here, we show that archaea responded to nanosilver-stress by expressing export enzymes, but were nonetheless still present at decreased abundance. While other studies clearly suggest that nanosilver affects the bacterial contribution to nitrification, our results indicate a greater impact in the maize rhizosphere on the archaeal involvement in this important ecological process.

### Direct and indirect effects on maize likely lead to hormesis

**Fig. 7:**
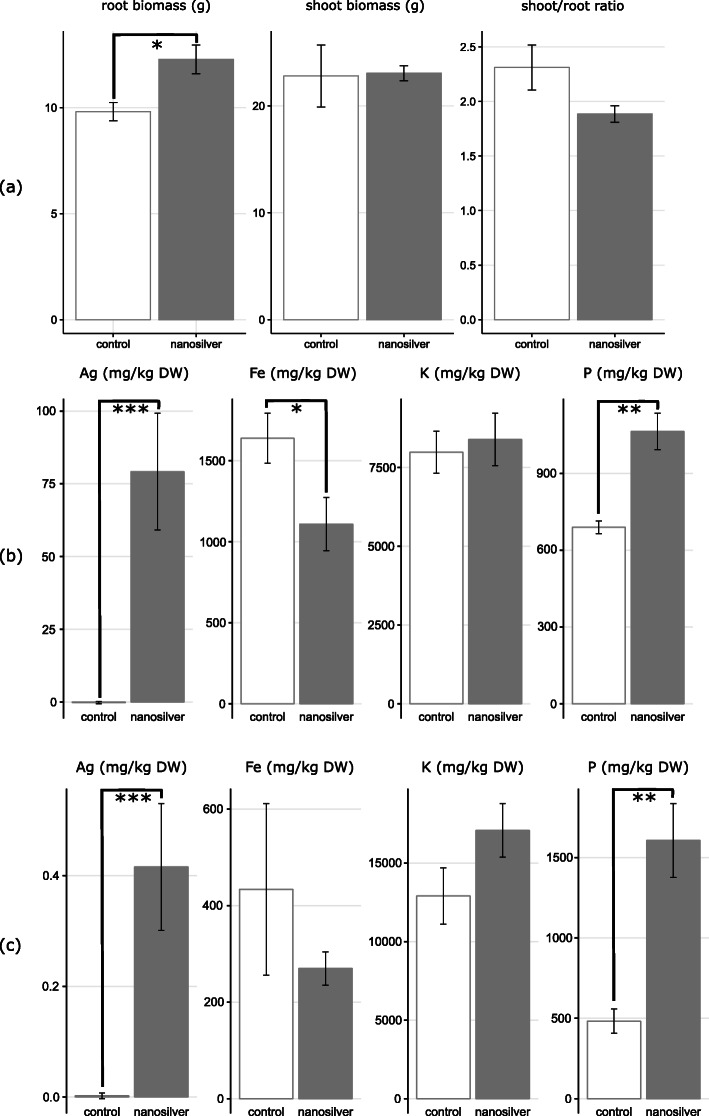
Maize biomass, nutrient, and silver concentrations in response to nanosilver exposure. Maize (**a**) root and shoot biomass and shoot/root ratio, (**b**) root, and (**c**) shoot concentrations of Ag, Fe, K, and P. Plants were grown for 117 days in soil with or without 100 mg kg^−1^ silver nanoparticles (20 nm). Values ± SE; *p < 0.05, **p < 0.01, ***p < 0.001

Plant roots are influenced by a large number of biotic and abiotic factors by virtue of their presence in soil. It appears that both of these types of factors contribute to the overall effect of nanosilver on maize. Silver, either in nanoparticle or ionic form, may be taken up by the plant and some fraction will be translocated to aboveground tissues [38]. Our data show that the average Ag concentrations in the roots and shoots of nanosilver-exposed plants were 79 mg kg^−1^ (dry biomass) and 0.42 mg kg^−1^, respectively, while values for the non-exposed plants were nearly non-detectable (Fig. 7). The low translocation factor results in low silver concentrations in the shoot, but silver concentrations in the roots are clearly high enough to cause stress. Elevated numbers of glutathione metabolism transcripts (Table 1) indicate the reaction of the plant to a disturbance in oxidative balance [39]. Another indication of a changing oxidative balance in maize is the higher abundance of germin transcripts. Germin and germin-like proteins have been linked to a wide range of functions, including a role as oxalate oxidase or as superoxide dismutase [40]. Superoxide dismutases are directly involved in defense against oxidative stress, while oxalate oxidases produce H_2_O_2_ which triggers signaling pathways that upregulate expression of genes for antioxidant enzymes [41].

In addition to these oxidative balance disturbances, aquaporin activity in maize seems to be significantly affected by exposure to nanosilver; specifically, transcripts of the aquaporin PIP are present at significantly increased abundance. Several in vitro studies with *Arabidopsis* seedlings [42] and rice seeds [43] have shown that nanosilver can activate aquaporin gene expression. Here, we show that nanosilver in soil exerts a similar affect. The mechanism behind this response is unclear, although it has been reported that silver can inhibit aquaporin activity through interaction with sulfhydryl groups of the proteins [44]. The increased aquaporin PIP transcripts may be a response of the plant to the inhibition of these important water channels.

Nanosilver exposure seems to also stress maize indirectly by increasing the abundance of potentially phytopathogenic fungi. A possible defense response of maize to phytopathogen infection can be seen in the induced transcription of genes involved in the production of beta-glucosidases and phenylpropanoids. Although not definitive, these findings could indicate a significant additional burden on maize grown in nanosilver-containing soil. Nonetheless, the findings certainly demonstrate that maize responds at the molecular and biochemical level to nanosilver exposure. The diversity of these physiological responses is also illustrated by changes in plant gene expression related to phytohormones and nutrient balance (Table 1). Plant hormone signal transduction is critical to many plant growth and development processes, and modulation of transcript abundance is likely indicative of the biomass changes that were noted upon nanosilver exposure. The increases in iron and phosphate transporter transcripts demonstrate nanosilver-induced modulation of maize nutrient balance. ICP analysis of maize tissues confirmed this (Fig. 7). While Fe concentration was significantly decreased (*p* = 0.041) in roots under nanosilver exposure from 1683 to 1115 mg kg^−1^, root P concentration significantly increased (*p* = 0.002) from 692 to 1053 mg kg^−1^ and root K concentration showed no effect with values stable around 7800 mg kg^−1^. A different pattern emerged in the shoots; although the increased P concentration was still evident (*p* = 0.003) with values of 1612 versus 494 mg kg^−1^, the statistical significance of the Fe decrease was lost, although a decrease from 435 to 283 mg kg^−1^ was evident. In addition, the K concentration exhibited a statistically non-significant trend of increase (*p* = 0.12) from 1290 to 1725 mg kg^−1^. These changes in nutrient content could be attributed to a variety of causes, including a direct molecular effect of nanosilver on these enzymes or indirectly as a plant response to physiological changes from the exposure.

Overall, it is clear that there are a number of diverse ways through which nanosilver directly or indirectly affects maize, and ultimately this seems to induce an increase in root biomass (Fig. 7). Maize plants exposed to silver nanoparticles in soil showed a significantly higher root biomass (*p* = 0.046) than the control plants, increasing from 9.8 g to 12.3 g. The shoot biomass was approximately 23 g and remained unaffected by exposure; the shoot/root-mass ratio decreased from 2.3 to 1.9, although the effect was not statistically significant. Although most studies on nanosilver exposure highlight a neutral to negative effect on plant growth, our results are not the first indication of a nanosilver-induced plant biomass increase [45]. We did not observe an increased presence of any plant-growth-promoting factors such as plant-growth-promoting rhizobacteria. Therefore, it seems that the above-described direct and indirect impacts of nanosilver on maize may result in a hormetic response of the plant. Hormesis is an “umbrella term” used to describe instances where low doses of generally toxic substances can stimulate biological systems [46]. In plants, this phenomenon can arise through a variety of mechanisms, including both substrate interactions and activation of defense reaction pathways that are involved in multiple physiological processes [47]. It has been reported that low concentrations of arsenic can increase the bioavailability of inorganic phosphate through competitive interactions related to soil adsorption [48]. Hence, arsenic at low doses can increase plant growth through enhanced P availability and uptake. Although a similar mechanism cannot be proposed for silver, the increased P concentrations measured in exposed maize tissues could be involved in the biomass increase. In addition, reactive oxygen species (ROS) and antioxidant molecules and enzymes play central roles in plant defense. We found increased transcript abundances for genes involved in glutathione and germin metabolism. These molecules play important roles in ROS signaling and scavenging and hence, could initiate mechanisms which account for the hormetic effect. For example, in wheat, it has been shown that long-term exposure to low Cd concentrations in soil can have a hormetic effect, being linked to a reduction of the ROS level and an increase in the activity of glutathione reductase [49]. In addition, hormesis potentially could also arise through cross-talk interactions between metals and biotic stress caused by phytopathogens. This is possible because metal ions can evoke the production of secondary metabolites that are involved in defense against pathogens, including molecules such as flavonoids, among others [47]. Hence, it is possible that the increased phenylpropanoid transcript levels here are caused by silver and subsequently elevate the protection level of maize against phytopathogens, inducing a hormetic effect. Importantly, the cause of the increased maize root biomass under nanosilver-exposure cannot be determined with certainty, but a wide range of processes, covered by the “umbrella term” hormesis, seem likely to have played a role here.

## Conclusions

Using the extensive information, the rhizosphere metatranscriptome has to offer, we show that soil borne nanosilver has various unintended effects on maize and its rhizosphere microbiome. On the prokaryote side, nanosilver can interfere with the nitrogen cycle due to a decrease in abundance of archaea taking place in the process and can potentially compromise natural biocontrol systems because of a decrease in abundance of bacterial groups with a biocontrol function. This latter effect can yield an increase in phytopathogen activity, instead of the intended decrease. Nanosilver also induces direct stress on the maize plants, likely through oxidative stress and aquaporin interference. Together, these indirect and direct effects on maize suggest that the observed increase in root biomass, which could be considered a positive outcome, is the result of hormetic growth stimulation that is unlikely to be sustainable in the long term. Hence, the overall balance suggests that the application of nanosilver in agriculture comes with significant unintended effects which could turn out to be negative for crop productivity and ecosystem health in the longer term. Therefore, it seems essential that these microbiome-related processes are included when assessing the risk associated with nanosilver use in agriculture.

## Methods

### Material and experimental set-up

Uncoated silver nanoparticles (99.99% purity, 20 nm diameter) were obtained in solid form from US Research Nanomaterials, Inc., Houston, TX, USA. Particle zeta potential and hydrodynamic size were characterized in 500 mg/L solutions by dynamic light scattering (DLS) on a zetasizer (Malvern Zetasizer, Nanoseries ZS90). The average size for AgNP is 259.7 nm (± 10.05), and the zeta potential is − 30.3 mV (± 2.71). The particles were also characterized by transmission electron microscopy (TEM) (Hitachi HT7800). TEM indicated a wide variety of sizes due to clustering of the original particles of ca. 20 nm (Additional file 1). Soil was collected from the top 30 cm of an agricultural corn field in Diepenbeek, Belgium (50°56′05.3′′ N 5°24′41.2′′ E) and was characterized as sandy loam (55% sand, 30% silt, 15% clay) with a pH of 6.98, an electrical conductivity (EC) of 335 μS cm^−1^ and an effective cation-exchange capacity (CEC) of 20.7 meq/100 g. After collection, the soil was sieved to 6 mm and homogenized. *Zea mays* variety LG 30.223 seeds were acquired from LimaGrain Belgium. Soil nutrient content was augmented by fertilization until conditions favorable for maize cultivation were reached: 106 mg N g^−1^ soil, 34 mg P g^−1^ soil, 31 mg K g^−1^ soil, and 15 mg Mg g^−1^ soil. Maize plants were grown individually in 10 L-pots, each pot containing 10 kg of soil. Three replicate pots contained nanosilver at 100 mg kg^−1^, which was added by mechanical mixing for 5 min. This nanosilver concentration was established in earlier research, because it provides a baseline response in the system and takes into account the possibility of nanosilver accumulation in agricultural soils due to the application of biosolids and nano-enabled agrichemicals [45]. Three other replicate pots were not amended with nanosilver and were used as controls. Before planting, maize seeds were soaked in tap water overnight. All pots with seeds were randomly placed in a climate chamber with the following conditions: 12 h daylight photoperiod, a temperature cycle of 22 °C/18 °C, and a relative humidity of 50%. After 117 days, all plants were harvested and soil samples from all conditions were taken for metatranscriptomic analysis. Samples of the rhizosphere soil, operationally defined as the soil that remains attached to the roots after light shaking, were flash-frozen in liquid nitrogen and stored at – 80 °C.

### Plant biomass and element content analysis

At harvest, the wet and oven-dry biomass of root and shoot tissues were determined. For plant element content analysis, oven-dried samples of roots and shoots (approximately 0.5 g) were digested in 50 ml polypropylene digestion tubes with 5 ml of concentrated nitric acid at 115 °C for 45 min using a hot block (DigiPREP System; SCP Science, Champlain, NY, USA). A small volume of H_2_O_2_ was included to ensure complete digestion. The resulting digests were analyzed for Ag, Fe, K, and P using inductively coupled plasma optical emission spectroscopy (ICP-OES; iCAP 6500 Thermo Fisher Scientific). Samples with Ag content below the ICP-OES limit of quantification were subsequently analyzed by Inductively Coupled Plasma-Mass Spectrometry (ICP-MS; Agilent 7500ce).

### Soil RNA extraction and sequencing

Total RNA was extracted from the rhizosphere soil samples using the PowerSoil® RNA Isolation Kit (Mo Bio Laboratories Inc., CA, USA), according to the manufacturer instructions. Starting material for every extraction was 6 g of soil. Extracted RNA was additionally purified using the NucleoSpin® RNA kit (Macherey-Nagel, Düren, Germany), including DNase-treatment according to the manufacturer’s instructions. RNA integrity, purity, and concentration were confirmed by Experion^TM^ RNA assays (Bio-Rad Laboratories, Munich, Germany); these data were used as an indicator for the need to repeat failed extractions.

Sequencing was performed by Macrogen (Seoul, South Korea). Libraries were constructed using the Illumina TruSeq Stranded Total RNA kit (Illumina, USA) without rRNA depletion or mRNA enrichment. Illumina HiSeq4000 paired-end sequencing (2 × 100 bp) resulted in at least 35 million high-quality reads per library (Table 2). The raw sequencing data were deposited at the European Nucleotide Archive (ENA) under the project ERP024369.

**Table 2:** Basic metatranscriptome composition

Treatment	Sample	Total high quality reads	Read proportions			
			rRNA reads		Non-rRNA reads	
			Number of reads	Percentage of total	Number of reads	Percentage of total
Nanosilver-exposed	Sample 1	35493754	33642151	94.8	1851603	5.2
	Sample 2	36666638	34699832	94.6	1966806	5.4
	Sample 3	35067906	33332673	95.1	1735233	4.9
Control	Sample 1	43534310	41955731	96.4	1578579	3.6
	Sample 2	44648348	42845994	96	1802354	4
	Sample 3	35339870	34033583	96.3	1306287	3.7

Basic composition of the metatranscriptomes obtained from maize rhizosphere soil with or without 100 mg kg^−1^ silver nanoparticles after 117 days of growth and exposure.

### Metatranscriptome analysis

Raw sequencing reads were controlled for quality using FastQC version 0.10.1 (Andrews, 2010, available online at http://www.bioinformatics.babraham.ac.uk/projects/fastqc). Based on the FastQC quality report, the Kraken pipeline [50] was used to remove potential adapter sequences, poor quality reads (Phred score < 10), short reads (< 30 nucleotides), and reads without a counterpart, all while concurrently maintaining read-pairing during processing. The resulting high-quality reads were used for taxonomic and functional analysis. Taxonomic analysis aimed at characterizing the communities of archaea, bacteria, and fungi through marker gene analysis, which was achieved through the One Codex platform that utilizes targeted loci including 5S, 16S, 23S, 18S, 28S, and ITS [51]. For functional analysis, the quality-filtered reads were sorted using SortMeRNA [52], which separated rRNA from non-rRNA and thus, potential mRNA. Sorting was performed with standard parameters, using the SILVA-16S-18S-SSURef_115_NR99 and SILVA-23S-28S-LSURef_115 databases [53]. FragGeneScan [54] version 1.17 with the Illumina 0.5% error model was used on the potential mRNA reads to filter out undesired sequences such as non-coding regions, leaving only putative genes. These remaining sequences were subsequently aligned to the NCBI-nr database with DIAMOND [55] version 0.8.38, applying an *E* value cut-off of 10^−3^. The resulting alignments were annotated to the InterPro2GO [56] and KEGG [57] databases through MEGAN6 [58] using default LCA parameters (min score: 50, top percent: 10, min support).

### Statistical analyses

R version 3.3.2 [59] was used for the statistical analyses. Parametric group mean comparisons such as Student’s *t* test and ANOVA were performed for the plant biomass and element content data.

The taxonomic community compositions were exported from One Codex for further analysis. Richness and Shannon diversity of archaea, bacteria, and fungi separately were calculated for the control and nanosilver-exposed conditions. Differences between the two treatments for these parameters were analyzed using *t* tests as parametric assumptions were fulfilled. Principal coordinates analyses (PCoA) based on Bray-Curtis dissimilarities were performed and plotted for the archaea, bacteria, and fungi communities individually, and the separation between the control and the nanosilver-exposed treatment was statistically evaluated by the use of analysis of similarity (ANOSIM). The relative abundance of individual taxa was analyzed for statistically significant differences between the two treatments (control vs. nanosilver-exposed) using the Bioconductor packages phyloseq [60] version 1.19.1 and DESeq2 [61] version 1.14.1 . The Wald test was used and statistical significances were considered if the adjusted *p* values < 0.05 (using the Benjamini and Hochberg procedure). Functional annotations extracted from MEGAN6 were statistically analyzed using DESeq2 version 1.14.1, also by means of the Wald test and adjusted *p* value < 0.05 (Benjamini and Hochberg) considered as statistically significant.

Network analysis of the taxonomic microbial community compositions was performed with the Cytoscape [62] version 3.4.0 plugin CoNet [63] by means of co-occurrence and co-exclusion interactions. Filtering was done by setting the minimum abundance per phylogenetic order to 40000 reads over all samples. Pair-wise associations between orders were calculated with the simultaneous use of Pearson and Spearman correlation, mutual information, Bray-Curtis, and Kullback-Leibler dissimilarity. The top and bottom 100 edges for each method were initially selected. Edges needed to be supported by at least two of these methods in order to be retained. Permutation was performed with 100 repetitions, and the resulting *p* values were used as a cut-off value at 0.01. The resulting network was visualized in Cytoscape.

## Supplementary information

**Additional file 1.** Transmission electron microscope image of the silver nanoparticles used in the study. Nanoparticles were obtained in solid form from US Research Nanomaterials, Inc. (Houston, TX, USA), and were applied to soil in this form.

## Data Availability

All raw metatranscriptome data are available at the European Nucleotide Archive (ENA) under the project ERP024369.
