# Supplementary material for: Nanoparticle treatment of maize analyzed through the metatranscriptome: compromised nitrogen cycling, possible phytopathogen selection, and plant hormesis
Source: Microbiome. 2020 Sep 9;8:127. doi: 10.1186/s40168-020-00904-y (PMC7488162; doi:10.1186/s40168-020-00904-y)

**Transmission electron microscope image of the silver nanoparticles used in the study**

Nanoparticles were obtained in solid form from US Research Nanomaterials, Inc. (Houston, Texas, USA), and were applied to soil in this form.


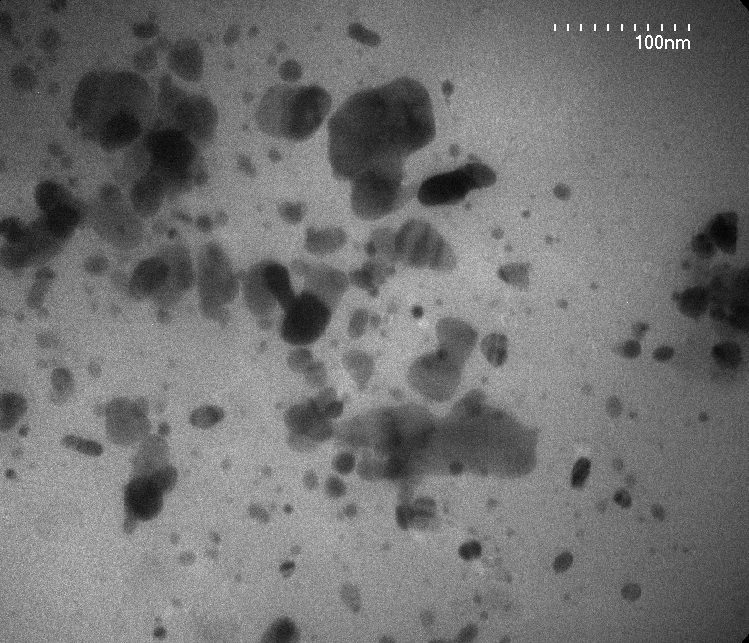

Supplement: Supplementary file 2 — Additional file 1. Transmission electron microscope image of the silver nanoparticles used in the study. Nanoparticles were obtained in solid form from US Research Nanomaterials, Inc. (Houston, TX, USA), and were applied to soil in this form. [file 40168_2020_904_MOESM1_ESM.docx]
